# Retraction: Alpha-2-Glycoprotein 1(AZGP1) Regulates Biological Behaviors of LoVo Cells by Down-Regulating mTOR Signaling Pathway and Endogenous Fatty Acid Synthesis

**DOI:** 10.1371/journal.pone.0215712

**Published:** 2019-04-16

**Authors:** The PLOS ONE Editors

Following the publication of this article [1], the following concerns were raised:

1. In Fig 5D the blank, control and experimental panels contain clusters of cells that appear similar across panels.
2. In Fig 3A, the GAPDH and S6K1 panels appear to contain black boxes below the bands.

The authors provided modified images for the panels in Fig 5D, which did not satisfactorily resolve the concerns raised. In addition, the authors provided primary data underlying all bar graphs, but the uncropped scans of blots and primary data underlying all other figures are unavailable.

In light of the above-mentioned concerns that question the validity of the study’s findings and owing to the lack of availability of data underlying the figures for which concerns were raised, the *PLOS ONE* Editors retract the article.

LC, XT, YL, MJ, PW, and PH agreed with the retraction.
